# A76 MEDICATION ADHERENCE AND BELIEFS ABOUT MEDICATION AND THE CORRELATION WITH KNOWLEDGE ABOUT INFLAMMATORY BOWEL DISEASE (IBD) IN PREGNANCY

**DOI:** 10.1093/jcag/gwab049.075

**Published:** 2022-02-21

**Authors:** V Govardhanam, P Tandon, R Jogendran, V Huang

**Affiliations:** University of Toronto Faculty of Medicine, Brampton, ON, Canada

## Abstract

**Background:**

Inflammatory bowel disease (IBD) is a group of chronic inflammatory conditions including ulcerative colitis (UC), Crohn’s disease (CD) or IBD-unclassified that affect women of childbearing age. women with IBD have poor knowledge of disease management during pregnancy, as demonstrated by studies using the validated Crohn’s and Colitis Pregnancy Knowledge (CCPKnow) tool. These patients who lack knowledge about IBD and reproduction tend to have misguided fears and make uninformed decisions such as voluntary childlessness (VC) or poor medication adherence.

**Aims:**

We aim to understand the correlation between improved knowledge about IBD and Medication Adherence Report Scale (MARS) and Beliefs about Medication Questionnaire (BMQ)

**Methods:**

Adult women with IBD attending the pregnancy IBD clinic at the University of Alberta from 2014–2018 were enrolled. Each patient completed the Crohn’s and Colitis Pregnancy Knowledge (CCPKnow) questionnaire at baseline and after individualized education delivered at each clinic visit. BMQ and MARS Questionaries were completed using a 0–5 Likert scale at each visit. BMQ and MARS data were analysed using Wilcoxon signed ranks test by comparing pre-conception, intrapartum data (trimester) and post-partum scores. BMQ questions were classified under the BMQ Concerns category and BMQ necessity category for regression analysis.

**Results:**

A total of 117 patients were enrolled in this study. 55 patients with CD (47.1%) and 62 patients with UC (52.9%). Statistically, a significant change was noted when comparing the median Trimester BMQ scores to pre-conception BMQ scores. (Z of -2.667, p=0.008) and Median post-partum BMQ scores to Median pre-conception BMQ scores. (Z of -2.547, p=.011). Trimester BMQ Concerns data had a strong negative correlation with CPPKnow scores (Correlation Co-efficient -.528, p<0.05). Median Trimester MARS data had a strong positive correlation with CPPKnow scores (Correlation Co-efficient 0.644, p<0.05).

**Conclusions:**

Increased CPPKnow scores in patients were correlated with patients that were less concerned about IBD medication use and were noted to have higher medication adherence as reflected in the MARS scores post-intervention. A dedicated pregnancy clinic aimed at improving IBD and pregnancy knowledge in women would encourage greater adherence to IBD medication.

**Funding Agencies:**

None

**Table:** Wilcoxon Signed Rank Tests comparing BMQ and MARS Median scores. The 3 temporal time periods are Preconception, Trimester and Post conception

	Median Trimester BMQ value – Median preconception BMQ value	Median postpartum BMQ value – Median Trimester BMQ value	Median postpartum BMQ value – Median preconception BMQ value	Median Trimester MARS value – Median preconception MARS Value	Median Postpartum MARS Value – Median Trimester MARS Value	Median Postpartum MARS Value – Median preconception MARS Value
Z score	-2.667	-0.325	-2.547	-0.672	-1.822	-0.493
Asymp.Sig. (2-Tailed)	0.008	0.745	0.011	0.502	0.068	0.622

